# Expanding the Role of Primary Care in the Prevention and Treatment of Childhood Obesity: A Review of Clinic- and Community-Based Recommendations and Interventions

**DOI:** 10.1155/2013/172035

**Published:** 2013-04-28

**Authors:** Michaela Vine, Margaret B. Hargreaves, Ronette R. Briefel, Cara Orfield

**Affiliations:** ^1^Mathematica Policy Research, 955 Massachusetts Avenue, Suite 801, Cambridge, MA 02139, USA; ^2^Mathematica Policy Research, 1100 1st Street, NE, 12th Floor, Washington, DC 20002-4221, USA; ^3^Mathematica Policy Research, 220 East Huron Street, Suite 300, Ann Arbor, MI 48104-1912, USA

## Abstract

Although pediatric providers have traditionally assessed and treated childhood obesity and associated health-related conditions in the clinic setting, there is a recognized need to expand the provider role. We reviewed the literature published from 2005 to 2012 to (1) provide examples of the spectrum of roles that primary care providers can play in the successful treatment and prevention of childhood obesity in both clinic and community settings and (2) synthesize the evidence of important characteristics, factors, or strategies in successful community-based models. The review identified 96 articles that provide evidence of how primary care providers can successfully prevent and treat childhood obesity by coordinating efforts within the primary care setting and through linkages to obesity prevention and treatment resources within the community. By aligning the most promising interventions with recommendations published over the past decade by the Institute of Medicine, the American Academy of Pediatrics, and other health organizations, we present nine areas in which providers can promote the prevention and treatment of childhood obesity through efforts in clinical and community settings: weight status assessment and monitoring, healthy lifestyle promotion, treatment, clinician skill development, clinic infrastructure development, community program referrals, community health education, multisector community initiatives, and policy advocacy.

## 1. Introduction

The identification of effective strategies to address the prevention and treatment of childhood obesity is critical to improving the health of the US population. National data from 2009 and 2010 show that nearly one in three children in America is either overweight or obese, and the numbers are even higher among certain demographic groups [1]. In the short term, obesity poses significant risks for children's physical health and psychosocial well-being [2, 3]. In the long term, many of today's children will age into adulthood with obesity that began in childhood and will experience the negative health consequences associated with obesity as adults, such as type II diabetes [4]. Addressing the high prevalence of childhood obesity will require coordinated and collective efforts in multiple sectors and settings—government, health care, school, workplace, and community—that influence the food and physical activity environments in which children live [2, 5].

Primary care providers (PCPs), defined for purposes of this paper as physicians, physician's assistants, nurse practitioners, registered nurses working in a primary care setting (e.g., community health center), or clinicians working in a school-based health center setting, have important roles in meeting obesity prevention goals. Primary care providers have traditionally measured patients' heights and weights to assess growth, development, and body mass index (BMI) and treated obesity and health-related conditions, but there is a recognized need to expand these roles to include advocacy, modeling healthful behaviors in the community, and counseling individuals and families about obesity prevention [5, 6].

A number of scientific organizations have published recommendations or guidelines for primary care providers to address childhood obesity prevention and treatment (see Table 1). The most recent, by the Institute of Medicine (IOM) in its 2012 report “Accelerating Progress in Obesity Prevention,” includes the goal to “expand the role of health care providers, insurers, and employers in obesity prevention.” Health care providers have a role in each of the four strategies recommended by the IOM to achieve this goal:

1. Strategy 4-1: provide standardized care and advocate for healthy community environments;
2. Strategy 4-2: ensure coverage of, access to, and incentives for routine obesity prevention, screening, diagnosis, and treatment;
3. Strategy 4-3: encourage active living and healthy eating at work; and
4. Strategy 4-4: encourage healthy weight gain during pregnancy and breastfeeding and promote breastfeeding-friendly environments.

Recommendations by the White House Task Force on Childhood Obesity [7], the American Academy of Pediatrics [8, 9], the American Heart Association [10], and other health organizations have focused primarily on the health care provider's role of assessment and monitoring of BMI, encouraging and supporting recommendations for physical activity and healthy eating, and serving as positive role models for obesity prevention [11]. The Guide to Community Preventive Services recommended behavioral interventions to reduce screen time but noted insufficient evidence for provider-oriented interventions (e.g., provider education, feedback, or reminders) for obesity prevention and treatment [12].

### 1.1. Motivation for the Study

Despite these recommendations, PCPs are not doing as much as they should to prevent and treat childhood obesity. Data from a 2008 national survey of PCPs found that fewer than half of all PCPs assessed BMI percentiles regularly in children, and only 18% reported referring children for further evaluation or management [11]. Most (58%) reported never, rarely, or only sometimes tracking patients over time concerning weight or weight-related behaviors [11]. National survey data from 2007 found that 12% of physician office visits of all child or adult patients included counseling about nutrition or diet [13]. Obstacles limit the ability and activity of PCPs to meet these recommendations. Several studies identified a lack of office time to gather background information from families as a major impediment to addressing healthy weight [14–17]. Other obstacles include lack of awareness of the issue, lack of comfort or skill counseling families on the issue, need for organizational prompts, and lack of familiarity with available community resources for lifestyle counseling or obesity prevention programs [5, 11, 18–21].

Evidence suggests that with the right interventions and activities, PCPs can effectively play an expanded role in preventing and treating obesity among children and adolescents [20]. Obstetricians and gynecologists also play an important role in prenatal care, monitoring maternal weight gain, and encouraging and supporting breastfeeding [22]. The purpose of this review is to identify effective or promising practices in the expanded roles that are now recommended for PCPs (see Table 1). These roles include the following:

1. *weight status assessment and monitoring:* assessment and monitoring of BMI, nutritional intake, physical activity level, and other indicators of weight status in children and adolescents;
2. *healthy lifestyle promotion:* dissemination of healthy lifestyle recommendations and materials as part of primary prevention efforts in the primary care setting, excluding healthy lifestyle promotion that is part of patient treatment (item no. 3);
3. *patient treatment:* use of evidence-based techniques, such as behavioral and motivational counseling, within the primary care setting to treat patients identified as overweight or obese (treatment may include healthy lifestyle promotion);
4. *clinician skill development:* education and training on evidence-based assessment and counseling techniques;
5. *clinical infrastructure development:* implementation of capacity building within the primary care setting, such as improvements to organizational systems or care models used by providers;
6. *community program referrals:* referral of patients to community-based obesity treatment programs outside of the primary care setting;
7. *community health education:* dissemination of healthy lifestyle recommendations and materials as part of prevention efforts in the community setting;
8. *multisector community initiatives:* participation in multisector obesity prevention and treatment initiatives to achieve policy and systems goals; and
9. *policy advocacy.* support of and advocacy for policy changes in the broader community setting.

This review, guided by a socioecological framework and a systems approach [5, 23], focuses on the intersection between PCPs (including community health centers) and public health in the community. Studies of child or family interventions in primary care settings or community interventions with a direct link to primary care (e.g., a community intervention with active referral to PCPs) were the focus of the review. Although other reviews on childhood obesity and health care have been published since IOM's 2005 report on “Preventing Childhood Obesity,” the extent to which they summarize the specific roles of primary care providers in implementing the intervention varies [24–26]. This review updates the most recent reviews of literature on the primary care role in obesity prevention and treatment published from 2005 to 2012, addresses 2012 IOM recommendations that emphasize both a clinical and community advocacy role for PCPs [5], and incorporates multisector interventions and community advocacy-specific interventions involving PCPs. It also recognizes the new imperatives or opportunities to do more based on the Affordable Care Act changes requiring preventive care as an essential health benefit and eliminating cost sharing for preventive services [27].

## 2. Materials and Methods

**Figure 1:**
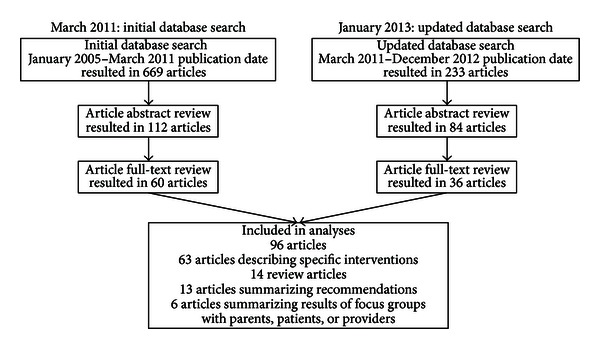
Literature review process.

We conducted a review of clinic- and community-based obesity interventions with a primary care component to identify evidence of effective roles of primary care in addressing the epidemic. The first phase of the scan was conducted in March 2011 (Figure 1). We searched for literature published from 2005 to 2011 using search terms listed in Table 2. Our search included PubMed and other databases (ESCO Academic Search Premier, Cochrane Central Register of Controlled Trials, ERIC, and Health Technology Assessments) and resulted in 669 articles. For these articles, we reviewed abstracts for relevance and to ensure that the intervention took place in the United States, which refined the list to 147 articles. We retained the articles that described an obesity intervention for children and/or families that took place in a primary care setting, a school health center, or a community setting (community health center; pediatrician; tribal health center; Special Supplemental Nutrition Program for Women, Infants, and Children (WIC) clinic; and so on) with some link to primary care. The elimination of unrelated school-based, policy, environmental change, and workplace interventions that did not include a link to the primary care setting, as well as articles that focused primarily on primary care recommendations (rather than primary care interventions), further refined the scan to 112 articles.

We reviewed the remaining 112 full-text articles to ensure that each article fits the original criteria and to document the findings. Of the full-text articles reviewed, 60 met the criteria. We updated the review in January 2013 for the literature published in 2011 and 2012. The additional review resulted in 36 articles that met our criteria, for a total of 96 articles considered in this paper. These sources included 63 articles describing specific interventions; 14 that reviewed existing interventions; 13 summarizing recommendations for the treatment and prevention of childhood obesity; and 6 that summarized the results of topic-related focus groups with parents, children, or clinicians. The full list of citations for articles considered in this review is available upon request from the authors.

## 3. Review Findings

The 96 articles that met the criteria for this review provide examples of how pediatricians, PCPs, and communities have implemented clinic- and community-based programs and initiatives targeting the prevention, screening, diagnosis, and treatment of obesity among children and adolescents. The interventions reviewed typically took place in a primary care clinic, pediatrician's office, community health center, school-based health clinic, university research program, WIC clinic, or other community setting. In this section, we summarize the paper findings regarding the efficacy of these efforts, when available, and describe how these interventions align with current recommendations for obesity prevention and treatment in the nine areas identified in Table 1. Because an article could address multiple primary care physician roles (e.g., weight assessment as well as treatment of obesity), articles can be cited in more than one category. Interventions reporting statistically significant improvements in child weight status are noted in Table 1. Methods used to assess changes in children's weight status include change in BMI; BMI *z*-score (i.e., the number of standard deviations of the value for an individual away from the mean value of the reference population); BMI percentile for age and gender; BMI velocity; kilograms or pounds lost; percent healthy weight, overweight, and/or obese; and waist and hip girth. The majority of interventions lasted between four and 12 weeks, and most follow-up efforts occurred over less than 12 months. 

### 3.1. Weight Status Assessment and Monitoring

Annual assessment of weight status through the use of BMI compared with age-sex BMI percentiles in growth charts in children and adolescents is widely recognized as a standard of care in the primary care setting [2, 9, 28]. Although a healthy weight assessment routinely involves some form of measuring body weight, evidence suggests that a complete assessment should also include indicators of healthy diet, active living, and child and family health history [8, 9]. Most interventions reviewed included an evaluation of the patient's overall health through patient and/or parent discussions or questionnaires in addition to assessment of weight status by use of BMI or growth charts. Methods used to assess or monitor weight included BMI, BMI *z*-scores, and comparisons to reference growth charts and standards for overweight and obesity. McKee et al. [16] integrated parent-completed questionnaires into routine primary care visits to assess family history of diabetes and cardiovascular disease, parents' height and weight, child's television and play habits, and child's intake of meals in front of the television; information collected from parents was used to inform weight assessment and guide the content of counseling and goal setting for overweight patients.

Recommendations state that PCPs should track annual BMI assessments over time to assist clinicians in recognizing major changes in weight relative to height [8, 9]. Two articles reviewed suggested that integration of BMI assessment into frequently used electronic medical record (EMR) systems or hand-held personal digital assistants could facilitate increased use of BMI as a screening tool and as a method of effectively tracking BMI over time for the purposes of monitoring [18, 20]. However, only one article reviewed specifically discussed integration of BMI collection into EMR systems. Savinon et al. [29] found that customized EMR templates designed to facilitate assessment of BMI and screening and counseling for overweight patients increased the frequency of children screened for BMI, as well as the diagnostic rate for overweight and obesity.

Articles reviewed suggest that multiple barriers might limit the assessment and monitoring of BMI in the clinic setting, including lack of familiarity with the use of BMI; lack of agreement about the utility of BMI as a screening and intervention tool; lack of office time to gather background information from families; and lack of practice-level resources conducive to simple, frequent use of BMI [11, 18–21]. Several articles noted the importance of familiarizing clinicians with weight assessment tools, including BMI assessment calculators, Centers for Disease Control and Prevention guidelines for BMI interpretation, and educational materials to increase uniformity of screening and improve clinician self-efficacy [20, 30–32]. Perrin et al. [20, 31] suggested that age-specific office-based tools may assist practitioners in communicating results of BMI assessment to families and in evaluating the patient's readiness to change. Another study that promoted use of BMI tools found a significant decrease in BMI at 5 months, but not 12 months, among children participating in a primary care-based program that combined clinician training in weight assessment with an eight-week, family-based behavioral intervention [33].

### 3.2. Healthy Lifestyle Promotion

Multiple recommendations suggested that promotion of healthy lifestyle behaviors such as adherence to recommended dietary guidelines, increased participation in physical activity, and limiting screen time and sedentary behavior should be incorporated into standard clinical practices for clinicians who serve children and adolescents [7–9, 12, 34–36]. These recommendations apply to both prevention and treatment of obesity in the primary care setting. Healthy lifestyle promotion as used in clinic-based treatment interventions is discussed in more detail in the following section; here, we describe notable examples of healthy lifestyle promotion as it pertains to the prevention of overweight and obesity in children and adolescents. We identified significantly fewer articles in this category.

The IOM suggests that providers utilize a multifaceted approach to patient education, recognizing that patients may have different learning styles, needs, and preferences [37]. Incorporation of healthy lifestyle promotion in the primary care setting may involve distribution or display of educational materials on nutrition, physical activity, and screen time in conjunction with verbal counseling of patients. Kubik et al. [15] described a prevention intervention that incorporates educational brochures on behavior-regulated activities, a Kid's Goal Board, and a Parent Tip Board in the waiting room area. Perrin et al. [20] suggested that PCPs should incorporate messages about healthy weight management, such as limiting screen time and sugar-sweetened beverages and increasing physical activity, into conversations with patients and parents during regular office visits. These conversations might be particularly important for children who are more likely to be overweight. Materials including healthy weight messages should be made available in multiple languages representative of the populations served by the clinic [38]. 

It is notable that recommendations by health organizations regarding reduced screen time have become more common in recent years [7, 12]. This is expected as screen time is a more recently accepted measure of inactivity (i.e., as one measure of physical activity) compared to more established measures such as healthy eating. It is also notable that, although several recommendations stated the need for PCPs to promote healthy weight gain during pregnancy, provide information and resources on breastfeeding, and promote guidelines for weaning children at the appropriate age [7, 9, 35, 36], few articles reviewed specifically addressed healthy lifestyle promotion among pregnant or breastfeeding mothers. Those that did [20, 21, 39] summarized recommendations for incorporating healthy lifestyle messages into prenatal or postnatal visits; however, none described specific health promotion interventions for pregnant or breastfeeding women in the primary care setting.

### 3.3. Patient Treatment

Although few health organization recommendations specifically addressed the role of the PCP in the treatment of overweight and obesity in children and adolescents [2, 12, 28], most of the articles reviewed that occurred in or were intended for the primary care setting involved interventions that were designed to treat the children who were identified as overweight or obese through BMI assessment. The format of the treatment and the intensity, frequency, and length of engagement with clinicians varied across studies. Many of the health organization recommendations on promotion of healthy lifestyle discussed in the previous section also apply to obesity treatment interventions; most of the interventions shown to be successful included promotion of improved nutrition and exercise habits and reduced screen time.

For children with a BMI above a specified percentile, treatment interventions that incorporated individual case management or patient-centered counseling as a means for achieving a child's healthy weight showed some evidence of success. Examples of individual case management include private, age-appropriate conversations with clinicians regarding achieving healthy weight; goal setting; motivational interviewing; and conversations with registered dieticians about patient readiness, diet, and exercise. Of the seven studies in this category, six measured positive results—including weight loss, improved lifestyle habits, or increased parent confidence using provider recommendations—after patients participated in multiple individual sessions with the providers [15, 40–45]. Successful studies emphasized the need for providers to engage the patient in a dialogue about lasting lifestyle changes and the benefits of training clinicians on how to address ambivalence about making behavioral changes. The tone and language used to communicate messages regarding obesity and being overweight is important. Two articles discussed strategies that PCPs could use to deliver diagnosis and treatment options. In focus groups, parents expressed preferences for health care providers to communicate using clinical terms to explain the rationale for their concern and to provide specific treatment recommendations [46, 47].

When a treatment plan is established for an individual, many primary care practices sponsor or refer patients to interventions that provide group classes or activities to support individuals and families. Content of primary-care-based group interventions was diverse, including in-person physical activities, educational grocery store visits, interactive nutrition and exercise sessions, family cooking courses, and group discussions. Though the PCP might make the initial referral to interventions of this type, his or her role in the actual group treatment intervention was less clear from the articles reviewed. Dalton et al. [48] described a group-based intervention for parents using the National Institute of Health's We Can! curriculum, which is facilitated by PCPs; McClaskey [49] described a community-health center intervention involving group nutrition and physical sessions led by physicians. However, dieticians, interventionists, or nurses carried out the majority of primary care-based group interventions.

Multiple recommendations from health organizations cited the role that PCPs can play in educating parents about healthy eating, physical activity, and reduced screen time [7–9, 35, 36]. Kwapiszewski and Lee Wallace [43] found it critical to have full support from all intervention partners (providers, parents, and children) in commitment to lifestyle changes to treat obesity. Most interventions in both the patient-based or group format involved some form of parent involvement, with parents present during individual or group counseling sessions with a PCP or in attendance at parent-only meetings with a focus on goal setting, modeling healthy behaviors, or nutrition and/or physical activity decision making.

### 3.4. Clinician Skill Development

Recommendations suggest that in order to effectively prevent, diagnose, and treat obesity and overweight in children and adolescents, clinicians must be adequately trained in standardized, evidence-based assessment and counseling techniques [7]. Moreover, clinicians must be comfortable communicating results of weight assessment and monitoring to patients and their families. Haemer et al. [50] suggested that trainings that include the full spectrum of care, rather than weight assessment alone, might be more effective in improving efficacy, as providers could be more likely to diagnose a child as overweight or obese when they have the tools and comfort level to provide counseling and treatment.

Multiple articles reviewed suggested the need for physician training and decision support for use of techniques and tools for counseling on behavioral treatment approaches for childhood obesity [11, 24, 50, 51]. Seven of the articles reviewed involved training for physicians, nurses, and/or registered dieticians on the use of motivational interviewing techniques, goal setting for parents and children, and/or evidence-based tools for facilitating discussions on obesity. In most cases, training took place in person in a group format, though Stahl et al. [40] described a successful web-based training program for clinicians. Two studies described the results of provider education interventions. Clinicians trained on the use of a brief, structured intervention for school-age children involving the use of flash-cards and take-home games reported increased physician comfort and competence discussing obesity issues [52]. Pediatricians and registered dieticians who received training on motivational interviewing techniques reported the need for more role-playing activities and experience asking open-ended questions [53].

Web-based or in-person primary care clinician trainings for assessment of BMI, healthful eating, and active living habits among children and adolescents were found to be effective in increasing provider confidence in weight assessment [31, 40], rates of BMI assessment, and use of behavioral screening tools after at least a year [32, 38]. Trainings included reference charts for BMI and laboratory values, guidelines for discussion about healthy behaviors, and decision support charts. At least one training included guidance on assessment of parental readiness for change [31].

In 2004, the American Dietetic Association stated the need for PCPs to take into account regional and cultural differences when promoting healthy diet patterns among diverse populations and ethnic groups [34]. We examined five studies that took actions to make interventions culturally competent. Examples of ways that interventions took the cultural, linguistic, or literacy needs of their subjects into account included providing materials and activities in multiple languages, offering recipes to groups that incorporate cultural food preferences, or tailoring materials to families who might have low literacy levels. Of the three studies that explicitly described their efforts to adapt obesity interventions to their population(s), three reported improved weight status among participants [54–56]. These studies used diverse means to adapt to the needs of their populations, which included Latino families (bilingual and bicultural project staff), ethnically diverse youth (traditional recipes), and families with low literacy levels (adapted educational materials). Results from a focus group with Latino parents suggest that, among this population, health messages can be especially well received coming from a trusted health care provider [46]. However, a culturally competent health educator might help to extend the benefits of an obesity treatment program beyond a brief encounter with a provider [57].

### 3.5. Clinical Infrastructure Development

Few recommendations cited the need for PCPs to advocate for systemic changes in clinical practices to promote screening, diagnosis, and treatment in the primary care setting. However, five of the articles reviewed focused specifically on an intervention that implemented some form of capacity building within the clinic setting, such as improvements to organizational systems or care models used by providers. Each identified structural gap in primary care services and implemented systemic solutions focused on reorganizing clinical care delivery. One such study evaluated the implementation of a patient-centered medical home system in a community health center and found positive outcomes in lifestyle changes, reduced BMI, and increased physical activity in the patients one year after implementation [58]. Another study evaluated the adoption of principles of continuous quality improvement and adult learning theory in the office environment; physicians found these tools helpful, which resulted in increased BMI screening documentation [59]. A third study assessed the effectiveness of integrating practice-based pediatric obesity prevention and treatment clinics within existing primary care settings; these clinics were shown to be helpful in improving nutrition status among obese children [60].

Several studies reviewed involved systems changes targeted at multiple primary care clinics or health care organizations. Pomietto et al. [38] described the Steps to Health King County (STEPS) initiative, which promoted clinic staff training and integrated clinic systems changes across three local health care organizations. This effort eventually grew to a larger program used throughout the state of Washington. The Maine Youth Overweight Collaborative sought to improve clinical decision support in 12 primary care sites; findings showed increased assessment of BMI and use of behavioral screening tools, as well as increased parental satisfaction with services [32].

### 3.6. Community Program Referrals

An activity frequently cited in journal articles is the physician's role in the identification and recruitment of children and families into obesity prevention or treatment interventions. Of the 38 articles reviewed that described community-based interventions, 14 reported physicians referring their patients to research studies (8 articles) or other community-based programs (6 articles). However, physician recommendations rarely called out this role, with one exception. In 2010, the White House recommended that PCPs and insurance companies connect pregnant women and new mothers to breastfeeding support programs [7].

The obesity interventions described in these articles were mostly family-based counseling and treatment programs, lasting from eight weeks to six months, including group education sessions for parents and children, home visits, follow-up telephone calls, automated messages, and/or other family-oriented activities. Some were branded with program names and set curricula, including Kids on the Geaux [61], Kids N Fitness [62], Healthy Kids Healthy Weight [63], ENERGIZE! [64], Smart Choices for Healthy Families [65], and Family Insulin Resistance Management—FIRM [66]. Of the 14 articles reviewed, nine reported positive outcomes, including weight loss or reductions in BMI scores [62–65, 67–71]. However, some study limitations included a small sample size [70] and a low participant retention rate [62, 65]. Factors that reportedly contributed to the success of the programs included the dosage of the intervention, the use of healthy eating strategies and behavior modification techniques, and family participation in physical activities [62].

Three of the articles assessed the value of the PCPs' referral role in the interventions. Pinard et al. [65] reported that, in the Smart Choices for Healthy Families program, physician involvement was seen as a valuable partnership. While physicians recognized the importance of referring patients to community-based programs that they did not have time to offer, program lay leaders saw the benefit of physician referrals, including improved behavior change among the provider's patients. Quattrin et al. [69] reported that parents perceived the obesity treatment program as an extension of their pediatrician's care because of the close partnership between the pediatrician and program trainers, the pediatrician's recommendation of the program, and followup with patients who were in the program. However, a third article compared physician referrals less favorably with other community program recruitment strategies. Because the number of physician referrals was not as high as expected, the program deployed additional recruitment methods, including the use of radio ads and posting of program flyers [72].

### 3.7. Community Health Education

Outside of their usual clinical role, PCPs have a unique opportunity to serve as role models, educators, and promoters of healthy lifestyle practices to their patients and other community residents. In 2005, the American Academy of Pediatrics encouraged physicians to make use of community-based resources outside of their traditional hospital and outpatient office settings to instruct residents on the effects of individual and community factors on child health status and to promote the well-being of all children in the community [35]. This might seem a natural role for physicians, extending their health promotion efforts with their patients to the community. Unfortunately, although many health care providers are aware of the childhood obesity epidemic, are concerned about its health impacts, and want to work on its prevention, they continue to see themselves primarily as clinical practitioners and not as health educators or advocates in the broader community [73].

PCPs can fill several roles in such community health education efforts by serving on leadership teams; providing advice on community messages; volunteering as institutional partners in the funding, planning, and evaluation of community awareness campaigns; and collaborating with community partners on marketing healthy food choices and physical activity. Of the articles reviewed, four focused on the physician's role in community-level obesity prevention initiatives. Health care providers served on the leadership team of the SWITCH program, which included a community awareness campaign to modify key health behaviors, increasing physical activity, improving nutrition, and reducing screen time [74]. PCPs also served on the community task force that led the Tioga County Fit for Life initiative, a comprehensive primary prevention program that used school-based health education classes, a virtual wellness club, and community health fairs to promote healthy nutrition and physical activity [75]. Two other studies reported that health care providers were involved in community initiatives that implemented the national We Can! program developed by the National Heart, Lung, and Blood Institute (although their roles were not specified) [76, 77].

Although it is important to note the participation of health care providers in these initiatives, outcomes were not reported in three of the four articles [74, 76, 77]. The fourth study, a five-year longitudinal analysis of grade-specific rates of overweight and obesity of participating children, showed that overweight and obesity rates increased in all cohorts. Factors cited for the program's failure included inadequate reach of key health messages and lag time between the messages' dissemination and uptake [75]. It is fair to assume that the involvement of PCPs in these initiatives was not responsible for their lack of reported success. In contrast, health care providers have played important roles in numerous more effective community interventions targeting both obesity prevention and treatment (see the next section).

### 3.8. Multisector Community Initiatives

Over the past decade, PCPs have been encouraged to build partnerships across disciplines to work collaboratively with public health departments and other colleagues, to identify and decrease barriers to the health and well-being of the children in their communities, and to coordinate and focus new and existing services for the benefit for all local children [34, 35]. In the articles we reviewed, health care providers participated in six multisector obesity prevention and treatment initiatives that achieved intermediate policy and systems goals [78–80]; changes in children's food and physical activity environments [80, 81]; and population-level health outcomes, including reduced BMI scores [82, 83] and changes in overweight and obesity prevalence trends [78, 79, 83].

Two projects used a multisector intervention model that started as a community-based research study at Tufts University. In Shape Up Somerville, 50 medical professionals were trained on childhood obesity guidelines and current BMI screening practices as part of a community-wide effort in Somerville, Massachusetts, to increase daily physical activity and healthy eating through programming, physical infrastructure improvements, and policy work [82]. North Carolina's Health Department patterned its Childhood Obesity Prevention Demonstration Projects after Shape Up Somerville. The state offered grants, training, technical assistance, and state-level partnerships and other resources to support local obesity prevention and treatment efforts in five counties. This included training PCPs to assess and treat childhood obesity in their communities [80].

PCPs were also involved in BMI assessment and treatment in community initiatives in Delaware and California. Delaware's 5-2-1-Almost None initiative targeted multiple sectors, including schools, child care providers, and primary care settings, to implement policy and practice changes, in addition to implementing a media-based social marketing campaign. PCPs promoted universal BMI assessment, preventive health messages, and early intervention and treatment of childhood obesity [78]. The California Endowment's Healthy Eating Active Communities program worked in six communities to prevent childhood obesity in five childhood environments—schools, after-school programs, neighborhoods, health care, and advertising. As part of the initiative, PCPs were trained on the importance of tracking BMI scores, delivering obesity prevention messages, linking families to community programs, and improving local nutrition and physical activity environments [81].

Two other communities included community-based BMI assessments in their multisector initiatives. In the Healthy Living Cambridge Kids program in Cambridge, Massachusetts, schools conducted BMI assessments and then referred students with high BMI scores to pediatricians for followup. The initiative included changes in city policies, implementation of a 5-2-1 community awareness messaging campaign, physical education enhancements in schools, food service reforms, family outreach, and farm-to-school-to-home programs [83]. In the Karanja research study, American Indian/Alaska Native tribes were randomly assigned to either a community-wide intervention that used five strategies—raising community awareness; providing health education; supporting behavior change; enhancing public health practice; and modifying local breastfeeding environments or policies to increase breastfeeding, limit consumption of sugar-sweetened drinks, and promote water consumption—or to an intervention that combined these community-wide activities with family-level interventions, including BMI assessment, counseling, and treatment. Health care providers conducted the BMI assessments in WIC clinics and maternal child health practices as part of routine visits [79].

Another promising initiative is the Healthy Weight Collaborative (HWC), a national quality improvement effort to share and spread promising and evidence-based practices to prevent and treat obesity among children [84]. In this learning collaborative, the National Initiative for Children's Healthcare Quality is working with about 50 community teams of primary care, public health, and community-based organizations to implement and test an integrated change package of strategies. These include (1) building a community coalition; (2) implementing a healthy weight messaging campaign; (3) conducting weight status assessments and follow-up plans; (4) integrating activities across community sectors; and (5) advocating for food and physical activity policy change. The HWC evaluation will be completed in 2013. 

Seven other studies in the review featured school-primary care partnerships or primary care interventions in school-based health centers. In four projects, nurses, nurse practitioners, and physicians in a school-based health center or WIC clinic offered counseling and treatment services to students identified with high BMI scores. The results of these programs were either not evaluated [85], minimal [14, 86], or mixed [87]. The other articles described school BMI assessment projects and a student walking project, whose outcomes were not evaluated.

### 3.9. Policy Advocacy

Several recommendations encourage health care professionals to support and advocate publicly for a number of policy changes, including increasing funding for childhood obesity prevention research; prioritizing capital improvement projects and school and community sports programs to increase opportunities for physical activity among students; and social marketing to promote healthful food choices, breastfeeding, and other healthy behaviors [2, 8, 9]. Although multisector community initiatives have used policy advocacy successfully to alter obesogenic community environments [88], one article reported on an initiative to increase public advocacy activity among PCPs [89]. Funded by the Robert Wood Johnson Foundation, the project sought to recruit, train, and reinforce 160 PCPs to become change agents and leaders in community advocacy to prevent childhood obesity. Physicians received a six-hour training using an advocacy resources guide. Posttraining surveys showed that the training had increased participants' comfort and motivation advocating publicly for healthy behaviors, including active living (26%), healthy eating (25%), breastfeeding (24%), and school and worksite policies (15%).

## 4. Conclusions

Identifying successful models that integrate primary care, public health, and community-based efforts is important to accelerating progress in preventing childhood obesity. This review aimed to identify the roles that PCPs play in childhood obesity prevention and treatment initiatives in the United States and, in doing so, to determine effective or promising strategies for primary care and community settings. The review, based on 96 peer-reviewed articles published from 2005 to 2012 that met study criteria, demonstrates that PCPs are increasingly being included in childhood obesity interventions, consistent with current recommendations from scientific and professional organizations. The review indicated an average of about 10 relevant articles published yearly during the period from 2005 to 2011 and nearly twice that number in 2012, supporting the increased attention to health care providers in the prevention of childhood obesity.

The rise in obesity among children indicates the need for new strategies that encompass more than individual-level behavior change or postassessment treatment. The prenatal and early childhood periods are critical times for growth and healthy lifestyle development. In the first two years of life, primary care pediatricians, WIC clinics, and community health centers have several opportunities during well-child visits to counsel parents about healthy lifestyles, to model healthful behaviors, and to refer families to community resources. Outside of their clinical role, primary care physicians can also serve as role models, educators, and promoters of healthy lifestyle practices and serve as leaders in community obesity treatment and prevention initiatives. However, national survey data on health practitioners and research studies suggest that PCPs continue to see themselves primarily as clinic-based practitioners and not as health educators or advocates in the broader community.

### 4.1. Study Limitations

Although this review identified nearly 100 articles addressing the topic, the ability to draw conclusions about the effectiveness of PCPs' roles in childhood obesity initiatives based on the review is limited by the lack of consistent reporting across studies about (1) specific PCP role(s) beyond referral and BMI assessment, (2) the level and duration of PCP involvement, and (3) child clinical outcomes or process outcomes. Interventions ranged from four to 12 weeks in duration, depending on the study and intervention methods. In addition, there was a general lack of long-term followup results; of the 20 interventions reviewed that had a significant impact on weight status, nine included followup over more than six months [33, 43, 54, 67, 70, 71, 79, 82, 83], and only three followed participants for more than one year [71, 79, 83]. In many cases, evaluations of the initiative were either not conducted or results were not reported. Interventions that did include an evaluation component used a range of outcome measures, including improved weight status, increased provider or parent knowledge, or increased rates of provider assessment of weight status or use of counseling tools, which made it difficult to compare the efficacy of results across articles reviewed. While change in weight or weight status was a frequently used outcome, the methods of weight assessment varied between interventions. Moreover, very few of the interventions reviewed utilized a randomized control study design, further limiting the ability to draw meaningful conclusions about the effectiveness of the interventions reviewed. For these reasons, the results of this review are primarily descriptive. 

While it is difficult to draw conclusions about the efficacy of the interventions considered due to the limitations mentioned previously, multisector community childhood obesity initiatives with primary care involvement were more likely to report positive outcomes than obesity initiatives in a single setting (school or clinic based). Multisector obesity prevention and treatment initiatives that achieved intermediate policy and systems goals included partnerships across disciplines, including PCPs, addressed children at all points along the prevention continuum, and used an ecological approach targeting individual, organization, system, and policy change. Positive outcomes included improvements in children's food and physical activity environments, reduced BMI scores, and changes in overweight and obesity prevalence. Successful models that integrated primary care, public health, and community-based efforts also shared several similarities:

- multisector messaging within a community;
- weight assessment training for clinicians;
- modeling of healthy behaviors for children (to reinforce their understanding of the concept);
- promotion of culturally competent approaches;
- parental involvement.

Because interventions of this type inherently involve multiple components, it is difficult to disentangle the roles to ascertain which individual components were especially successful or effective. Additionally, very few studies documented long-term effectiveness of interventions of this type, demonstrating a need for studies that measure the impact of multisector obesity initiatives over multiple years. Despite these limitations, this review provides a useful resource for PCPs, community organizers, researchers, and policymakers planning childhood obesity initiatives in their communities or primary care settings.

### 4.2. Next Steps

Future research on community-based childhood obesity interventions should collect and report information on the specific roles that PCPs played in the initiative, including the level of training and counseling skills, presence of role modeling, referrals to community resources, number and type of community partnerships, and public advocacy activity. Reporting on the process or implementation of the initiative as well as child-level and population-level outcomes will contribute to the evidence base for effective strategies by PCPs in the prevention and treatment of childhood obesity.

## Figures and Tables

**Table 1:** Recommendations for primary care providers for the prevention and treatment of obesity and selected citations.

Recommendation types (source)	Desired outcome					Citations for promising evidence-based interventions
	Active living	Healthy eating	Other health behaviors	Clinical practice	Community policy	
Weight status assessment and monitoring						

Primary care providers (PCPs) should screen children aged 6 and older for obesity (USPSTF 2010) [28].				×		Ewing et al., 2009^1^ [33] Kopp and Hornberger, 2008 [30] Kubik et al., 2008 [15] Mckee et al., 2010 [17] Perrin et al., 2007^2^ [20] Perrin et al., 2008 [31] Polacsek et al., 2009 [32] Savinon et al., 2012 [29] Van Gerwen et al., 2009^2^ [19]
PCPs should inquire about nutritional intake, calculate and plot BMI, and identify obesity-related comorbidities (AAP 2003) [9].		×		×		
PCPs should routinely track BMI and offer relevant evidence-based counseling and guidance about obesity prevention (IOM 2005) [2].				×		
PCPs should encourage parents to discuss weight status with their child's health care provider and monitor age- and gender-specific body mass index (BMI) percentiles (IOM 2005) [2].				×		
PCPs should calculate and plot BMI once a year in all children and adolescents and use change in BMI to identify rate of excessive weight gain relative to linear growth (AAP 2003) [9].				×		
PCPs should identify and track patients at risk by virtue of family history; birth weight; or socioeconomic, ethnic, cultural, or environmental factors. Recognize and monitor changes in obesity-associated risk factors for adult chronic disease, such as hypertension, dyslipidemia, hyperinsulinemia, impaired glucose tolerance, and symptoms of obstructive sleep apnea syndrome (AAP 2003) [9].				×		

Healthy lifestyle promotion						

PCPs should make AAP guidelines on screen time more available to parents, and young children should be encouraged to spend less time using digital media and more time being physically active (WH 2010) [7].	×					Kubik et al., 2008 [15] Perrin et al., 2007^2^ [20] Plourde, 2006 [39] Pomietto et al., 2009 [38] Waldrop and Ferguson, 2008 [21]
PCPs should inform pregnant women and women planning a pregnancy of the importance of conceiving at a healthy weight and having a healthy weight gain during pregnancy, based on the relevant recommendations of IOM (WH 2010) [7].				×		
PCPs should provide information to pregnant women and new mothers on breastfeeding, including the availability of educational classes, and connect pregnant women and new mothers to breastfeeding support programs to help them make an informed infant feeding decision (WH 2010) [7].			×			
PCPs should use behavioral interventions aimed at reducing screen time based on sufficient evidence of effectiveness for reducing measured screen time and improving weight-related outcomes. Interventions can be single component or multicomponent and focus on changing screen time through classes aimed at improving children's or parents' knowledge, attitudes, or skills (Community Guide 2011) [12].	×					
PCPs should promote healthy eating (AAP 2006) [8].		×				
PCPs should encourage parents to limit sedentary activity and make physical activity and sport recommendations to parents and caregivers that are consistent with the developmental level of the child (AAP 2006) [8].	×					
PCPs should recommend parent, guardian, and caregiver responsibilities for children's nutrition: (1) control when food is available, (2) provide social context for eating, (3) teach about food and nutrition when cooking and at the grocery store, (4) counteract inaccurate information from the media and other influences, (5) teach other caregivers what parents want their child to eat, (6) serve as role models and lead by example, and (7) promote and participate in regular daily physical activity (AHA 2006) [36].	×	×	×			
PCPs should promote guidelines for improving nutrition in young children: (1) parents choose mealtimes, not children; (2) provide a wide variety of foods; (3) pay attention to portion sizes; (4) use nonfat or low-fat dairy products; (5) limit snacking; (6) limit sedentary behaviors; (7) allow self-regulation of total caloric intake in the presence of normal BMI; and (8) have regular family mealtimes (AHA 2006) [36].		×			
PCPs should promote guidelines for nutritional quality after weaning: (1) delay the introduction of juice until at least 6 months of age, (2) respond to satiety cues and do not overfeed, and (3) include healthy foods and continue offering if initially refused (AHA 2006) [36].		×			
PCPs should promote breastfeeding for first nutrition and try to maintain it for 12 months (AHA 2006) [36].			×		
PCPs should support and promote healthful dietary patterns among diverse ethnic groups, taking into consideration regional and cultural differences (ADA 2004) [34].		×			
PCPs should support and promote (1) Dietary Guidelines for Americans for healthy children after the age of 2 years; (2) use of the US Department of Agriculture's Food Guide Pyramid as a guide for meeting dietary recommendations with use of the Food Guide Pyramid for Young Children aged 2 to 6; and (3) use of the Fitness Pyramid for Kids to encourage physical activity among children (ADA 2004) [34].	×	×			
PCPs should encourage parents and caregivers to promote healthy eating patterns by offering nutritious snacks, such as vegetables and fruits, low-fat dairy foods, and whole grains; encouraging children's autonomy in self-regulation of food intake and setting appropriate limits on choices; and modeling healthy food choices (AAP 2003) [9].		×			
PCPs should encourage, support, and protect breastfeeding (AAP 2003) [9].			×		

Patient treatment						

PCPs should offer or refer children aged 6 and older to intensive counseling and behavioral interventions to promote improvements in weight status (USPSTF 2010) [28].				×		Dalton et al., 2011 [48] Henes et al., 2010^1^ [44] Jacobson and Melnyk,2011^1^ [90] Jacobson and Gance-Cleveland, 2011^2^ [24] Kubik et al., 2008 [15] Kwapiszewski and Lee Wallace, 2011^1^ [43] Mcclaskey, 2010^1^ [49] Siegel et al., 2009^1^ [45] Stahl et al., 2011 [40] Taveras et al., 2011 [41]
PCPs should use technology-supported multicomponent coaching or counseling interventions intended to reduce weight on the basis of sufficient evidence that they are effective in improving weight-related behaviors or weight-related outcomes. The Task Force on Community Preventive Services recommends technology-supported multicomponent weight coaching or counseling interventions intended to maintain weight loss on the basis of sufficient evidence that they are effective in maintaining weight-related behaviors or weight-related outcomes. These interventions often also include other components, which can be technological or nontechnological (e.g., computers; videoconferencing; in-person counseling; written feedback; or computerized telephone system interventions that target physical activity, nutrition, or weight) (Community Guide 2011) [12].			×			
PCPs should offer pregnant women counseling, such as guidance on dietary intake and physical activity that is tailored to their life circumstances (IOM 2009) [22].	×	×		×		
PCPs should routinely offer relevant evidence-based counseling and guidance about obesity prevention (IOM 2005) [2].				×		

Clinician skill development						

PCPs should provide education and training in breastfeeding for all health professionals who care for women and children (SG 2011) [91].			×			Cluss et al., 2010 [56] Cronk et al., 2011^1^ [54] Haemer et al., 2011 [50] Holt et al., 2011 [51] Jacobson and Melnyk,2011^1^ [90] Maher et al., 2010 [57] McGaffey et al., 2011 [52] Perrin et al., 2008 [31] Polacsek et al., 2009 [32] Pomietto et al., 2009 [38] Stahl et al., 2011 [40] Schwartz et al., 2007^1^ [53] Savoye et al., 2011 [55]
Medical and other health professional schools, health professional associations, and health care systems should ensure that health care providers have the necessary training and education to effectively prevent, diagnose, and treat obese and overweight children (WH 2010) [7].				×	×	
Medical student, resident, and continuing medical education programs should consider and periodically review basic community pediatric competencies to be included in training and maintenance of certification efforts for pediatricians (AAP 2005) [35].					×	
Training programs and certifying entities should require obesity prevention knowledge and skills in their curricula and examinations (IOM 2005) [2].					×	
PCPs should foster communication by building partnerships across health-related disciplines and professional organizations and conduct effective nutrition education training programs for physicians, child nutrition personnel, and other health care providers on strategies that can be used with children to promote healthier eating habits (ADA 2004) [34].		×			×	

Clinical infrastructure development						

Hospitals and PCPs should use maternity care practices that empower new mothers to breastfeed, such as baby-friendly hospital standards (WH 2010) [7].			×		×	Anand et al., 2010 [58] Ariza et al., 2009 [59] Ariza et al., 2012 [60] Pomietto et al., 2009 [38] Polacsek et al., 2009 [32] Whitlock et al., 2008 [92]
PCPs should use interventions during pregnancy and after birth to promote and support breastfeeding (USPSTF 2010) [28].				×		
Insurers and accrediting organizations should provide incentives for maintaining healthy body weight and include screening and obesity prevention services in routine clinical practice and quality assessment measures (IOM 2005) [2].					×	

Referrals to community programs						

PCPs should educate themselves concerning the availability of community resources that affect the health and well-being of the children they serve (AAP 2005) [35].					×	Dreimane et al., 2007^1^ [62] Estabrooks et al., 2009^1^ [67] Foster et al., 2012^1^ [68] Heinberg et al., 2010^1^ [63] Paul et al., 2011^1^ [64] Pinard et al., 2012^1^ [65] Quattrin et al., 2012^1^ [69] Stark et al., 2011^1^ [70] Taylor et al., 2005^1^ [71]
PCPs and insurance companies should provide information to pregnant women and new mothers on breastfeeding, including the availability of educational classes, and connect pregnant women and new mothers to breastfeeding support programs to help them make informed infant feeding decisions (WH 2010) [7].			×			

Community health education						

PCPs should make AAP guidelines on screen time more available to parents, and young children should be encouraged to spend less time using digital media and more time being physically active (WH 2010) [7].	×					Agrawal et al., 2012 [77] Eisenmann et al., 2008 [74] Gombosi et al., 2007 [75] Moore et al., 2009 [76]
Education and outreach efforts about prenatal care should be enhanced through creative approaches that take into account the latest in technology and communications (WH 2010) [7].				×		
PCPs should become involved in the education of residents and medical students in community settings. Pediatricians have the unique opportunity to model roles outside the traditional clinical roles that students and residents encounter. Pediatric academicians should use resources from the AAP and the Ambulatory Pediatric Association to engage community pediatrician as educators, both in the care of individual patients in community-based practice and in roles related to promotion of the well-being of all children in the community. Community-based resources outside the bounds of the traditional hospital and outpatient office setting should be used to instruct residents on the effect of the community on child health status and the positive effect of interdependent collaboration of community agencies with health professionals on child health (AAP 2005) [35].					×	

Multisector community initiatives						

Local health departments and community-based organizations, working with health care providers, insurance companies, and others should develop peer support programs that empower pregnant women and mothers to get help and support from other mothers who breastfeed (WH 2010) [7].			×		×	Multi-sector: Chang et al., 2010 [78] Chomitz et al., 2010^1^ [83] Cousins et al., 2011 [80] Economos et al., 2007^1^ [82] Karanja et al., 2010^1^ [79] Samuels et al., 2010 [81] School-PCP: Edwards, 2005 [87] Stephens et al., 2011 [85] Tyler and Horner, 2008 [86] Whaley et al., 2010 [14]
PCPs should ensure that maternity care practices throughout the United States are fully supportive of breastfeeding and develop systems to guarantee continuity of skilled support for lactation between hospitals and health care settings in the community (SG 2011) [91].			×			
PCPs and other members of the community should interact and advocate to improve all settings and organizations where children spend time (e.g., child care facilities, schools, and youth programs). School and community resources should be considered as assets in developing strategies for the problems that children will face now and throughout their lives (AAP 2005) [35].					×	
PCPs should become comfortable with an interdisciplinary collaborative approach and advocacy effort to child health. Pediatricians can play an important role in coordinating and focusing new and existing services to realize maximum benefit for all children (AAP 2005) [35].					×	
PCPs should work collaboratively with public health departments and colleagues in related professions to identify and decrease barriers to the health and well-being of children in the communities they serve (AAP 2005) [35].					×	
PCPs should foster communication by building partnerships across health-related disciplines and professional organizations. Conduct effective nutrition education training programs for physicians, child nutrition personnel, and other health care providers on strategies that can be used with children that promote healthier eating habits (ADA 2004) [34].		×			×	

Policy advocacy						

PCPs should use community data (epidemiologic, demographic, and economic) to increase their understanding of the health and social risks on child outcomes and of the opportunities for successful collaboration with other child advocates (AAP 2005) [35].					×	
Include basic support for breastfeeding as a standard of care for midwives, obstetricians, family physicians, nurse practitioners, and pediatricians, and ensure access to services provided by International Board Certified Lactation Consultants (SG 2011) [91].			×			
PCPs should advocate for the appropriate allocation of funding for quality research in the prevention of childhood obesity (AAP 2006) [8].					×	
PCPs should advocate for (1) a school curriculum that teaches children and youth the health benefits of regular physical activity; (2) comprehensive community sport and recreation programs; (3) reinstatement of compulsory, quality, and daily physical education (PE) classes in all schools taught by qualified, trained educators; (4) provision of a variety of physical activity opportunities in addition to PE; and (5) development and implementation of a school wellness counsel on which local physician representation is encouraged (AAP 2006) [8].	×				×	Mayer, 2009^2^ [88] McPherson et al., 2012 [89]
PCPs should advocate the AHA 2006 Diet and Lifestyle Goals for Cardiovascular Disease Risk Reduction: consume an overall healthy diet, aim for healthy body weight, and encourage regular physical activity (AHA 2006) [8].	×	×			×	
PCPs should advocate for the development and implementation of a school wellness counsel on which local physician representation is encouraged (AAP 2006) [8].					×	
PCPs should work with local governments to change their planning and capital improvement practices to give higher priority to opportunities for physical activity (IOM 2005) [2].	×				×	
PCPs and other members of the community should interact and advocate to improve all settings and organizations in which children spend time (e.g., child care facilities, schools, and youth programs). School and community resources should be considered as assets in developing strategies for the problems that children will face now and throughout their lives (AAP 2005) [35].					×	
PCPs should become comfortable with an interdisciplinary collaborative approach and advocacy effort to child health. Pediatricians can play an important role in coordinating and focusing new and existing services to realize maximum benefit for all children (AAP 2005) [35].					×	
PCPs should support and advocate for social marketing intended to promote healthful food choices and increased physical activity (AAP 2003) [9].	×	×			×	

AAP: American Academy of Pediatrics; ADA: American Dietetic Association; AHA: American Heart Association; Community Guide: The Community Guide to Preventive Services; IOM: Institute of Medicine; SG: Surgeon General's Call to Action to Support Breastfeeding; USPSTF: United States Preventive Services Task Force; WH: White House Task Force on Childhood Obesity; see the list of references for complete citation.

^1^Indicates article describing an intervention that had a statistically significant effect on participants' weight or weight status.

^2^Indicates review article.

**Table 2:** Literature review search terms.

Search term		Search term		Search term		Search term
Healthy weight OR	AND/OR	Child OR	AND	Intervention OR	AND	Community intervention OR
Overweight OR		Family OR		Program OR		Community health services OR
Obesity OR		Families		Initiative OR		Primary health care OR
Weight-loss				Strategy OR		Tribe OR
				Strategies OR		Tribal OR
						Native American OR
						First Nation OR
						American Indian
						Indian Health Service OR
						Indigenous OR
						Islander OR
						Primary care OR
						Primary health care OR
						Community health

*Note*: search limits included humans, English, United States, and publication date from January 1, 2005, to December 31, 2012.
